# Vascular pathologies in chronic kidney disease: pathophysiological mechanisms and novel therapeutic approaches

**DOI:** 10.1007/s00109-021-02037-7

**Published:** 2021-01-22

**Authors:** Philip Düsing, Andreas Zietzer, Philip Roger Goody, Mohammed Rabiul Hosen, Christian Kurts, Georg Nickenig, Felix Jansen

**Affiliations:** 1 Heart Center, Department of Medicine II, University Hospital Bonn, University of Bonn, Venusberg-Campus 1, 53127 Bonn, Germany; 2 Institute of Experimental Immunology, University Hospital Bonn, University of Bonn, Venusberg-Campus 1, Bonn, 53127 Germany

**Keywords:** Chronic kidney disease, Atherosclerosis, Vascular calcification, Coronary artery disease

## Abstract

Cardiovascular disease (CVD) is a major cause of death in patients with chronic kidney disease (CKD). Both conditions are rising in incidence as well as prevalence, creating poor outcomes for patients and high healthcare costs. Recent data suggests CKD to be an independent risk factor for CVD. Accumulation of uremic toxins, chronic inflammation, and oxidative stress have been identified to act as CKD-specific alterations that increase cardiovascular risk. The association between CKD and cardiovascular mortality is markedly influenced through vascular alterations, in particular atherosclerosis and vascular calcification (VC). While numerous risk factors promote atherosclerosis by inducing endothelial dysfunction and its progress to vascular structural damage, CKD affects the medial layer of blood vessels primarily through VC. Ongoing research has identified VC to be a multifactorial, cell-mediated process in which numerous abnormalities like mineral dysregulation and especially hyperphosphatemia induce a phenotype switch of vascular smooth muscle cells to osteoblast-like cells. A combination of pro-calcifying stimuli and an impairment of inhibiting mechanisms like fetuin A and vitamin K-dependent proteins like matrix Gla protein and Gla-rich protein leads to mineralization of the extracellular matrix. In view of recent studies, intercellular communication pathways via extracellular vesicles and microRNAs represent key mechanisms in VC and thereby a promising field to a deeper understanding of the involved pathomechanisms. In this review, we provide an overview about pathophysiological mechanisms connecting CKD and CVD. Special emphasis is laid on vascular alterations and more recently discovered molecular pathways which present possible new therapeutic targets.

## Introduction

Chronic kidney disease (CKD) is defined as abnormality of kidney structure or function, present for more than 3 months. It is classified and staged based on cause, glomerular filtration rate (GFR G1-G5), and albuminuria category (A1-A3) [1]. Both albuminuria and reduced GFR have been shown to be associated with an increase in all-cause mortality which is especially driven by cardiovascular events [2, 3, 5]. Large meta-analyses have demonstrated that patients with impaired renal function have a 40–50% increased risk of developing coronary artery disease (CAD) compared to patients with normal renal function [4, 6]. This may, at least in part, be mediated by the fact that two of the most common causes for CKD, hypertension and diabetes mellitus, have also been identified as cardiovascular risk factors. However, even after adjustment for classic cardiovascular risk factors, CKD is still associated with an increased risk of coronary events, suggesting CKD to be an independent risk factor for CVD (Table 1) [6–8]. Furthermore, renal insufficiency correlates with the severity of coronary atherosclerosis and incidence of coronary events as well as mortality after myocardial infarction [7, 9, 10]. Rates of sudden cardiac deaths are increasing with declining renal function illustrated by a rate of 7 cardiac arrests per 100.000 hemodialysis sessions in the USA [11].

**Table 1:** Classical cardiovascular risk factors and CKD-specific risk factors fostering vascular disease

**Classical cardiovascular risk factors**	
Diabetes mellitus	
Hypertension	
Smoking	
Dyslipidemia	
Family history	
Age	
Male sex	
**CKD-specific cardiovascular risk factors**	
Vascular calcification	
Uremic toxins	
Oxidative stress	
Inflammation	

Vascular calcification (VC) is a common finding among CKD patients and even present in young adults with end stage renal disease (ESRD) lacking typical cardiovascular risk factors such as hypertension or dyslipidemia [12–14]. VC manifesting in the coronary arteries impairs coronary flow reserve and is associated with a marked increase in adverse cardiac events and cardiovascular mortality [13, 15, 16]. Interestingly, CKD affects CVD in a wider spectrum than ischemic heart disease alone. ESRD is also associated with aortic- and mitral-valve calcification, leading to a faster progression of valve stenosis and thus worse outcome for patients [17–19]. Furthermore, left ventricular hypertrophy, diastolic dysfunction, and cardiac fibrosis are known cardiac alterations which are strongly influenced by CKD [20]. Also, VC occurs more frequently in CKD patients, with a reported prevalence in dialysis patients greater than 80% [12, 21]. The underlying pathophysiological mechanisms for these multiple cardiovascular changes associated with CKD are not completely understood and therefore subject of ongoing research.

### Cardiorenal syndrome

An early step in attempting to establish a solid definition for the combination of CKD and CVD was taken in 2004 by the working Group of the National Heart, Lung, and Blood Institute in the USA. They proposed a first definition of “cardiorenal syndrome” (CRS) as an endpoint of cardiorenal dysregulation leading to an exacerbation of heart failure symptoms by an increased circulatory volume induced by kidneys and other circulatory compartments [22]. In 2008, this definition was extended by the consensus conference of the Acute Dialysis Quality Initiative into “disorders of the heart and kidneys whereby acute or chronic dysfunction in one organ may induce acute or chronic dysfunction of the other” [23]. They identified 5 subtypes of cardiorenal syndrome characterized by the order of the failing organ (cardiorenal versus reno-cardiac) and the temporal pattern (Table 2).

**Table 2:** Five subtypes of cardiorenal syndrome based on the consensus conference of the Acute Dialysis Quality Initiative. Modified after Ronco et al. [23]. *AHF*, acute heart failure; *ACS*, acute coronary syndrome

Type	Name	Definition
Type 1	Acute cardiorenal syndrome	Acute worsening of heart function (AHF-ACS) leading to acute kidney injury
Type 2	Chronic cardiorenal syndrome	Chronic abnormalities in cardiac function causing progressive chronic kidney disease
Type 3	Acute reno-cardiac syndrome	Abrupt worsening of renal function causing acute cardiac dysfunction
Type 4	Chronic reno-cardiac syndrome	Chronic kidney disease contributing to decreased cardiac function, cardiac hypertrophy, and/or increased risk of adverse cardiovascular events.
Type 5	Secondary cardiorenal syndrome	Systemic condition causing both cardiac and renal dysfunction.

This sub-classification of the cardiorenal syndrome represents a first important step in creating a clinically applicable definition in the common finding of combined cardiovascular and renal disease. However, it should be acknowledged that the pathophysiological background of this syndrome is more complex and extends beyond the heart and kidney alone. Recent investigations rather suggest a complex interaction of neurohormonal dysregulation, uremia, anemia and inflammatory pathways, endothelial dysfunction, atherosclerosis, and vascular calcification which make it challenging to precisely determine the sequence of pathophysiological events involved [24].

## Pathophysiology and therapeutic targets

### Chronic inflammation

Atherosclerosis and its manifestation in the coronary arteries, CAD, is supposed to be a key connector between CKD and cardiovascular morbidity and mortality. Atherosclerosis is characterized by a chronic inflammatory process of the vessel wall which is initiated through endothelial dysfunction [28]. CKD strongly correlates with CAD prevalence and progression and is believed to play a role in its pathogenesis as an independent risk factor [6–8]. CKD triggers vascular inflammatory processes, which is mirrored by an augmentation of inflammatory markers in the blood of CKD patients [25–27]. Elevated levels of inflammatory biomarkers such as C-reactive protein (CRP), interleukin-6 (IL-6), or tumor necrosis factor (TNF) have been shown to be associated with an increased risk of myocardial infarction and mortality [28–30]. While patients undergoing renal replacement therapy are exposed to inflammatory triggers owing to the invasive nature of the procedure, available evidence suggests that elevated inflammatory markers can be found in patients prior to initiation of dialysis [31–33]. In animal models, it was shown that oxidative stress, which is frequently observed in CKD patients, correlates significantly with an increase in inflammatory markers [34, 35]. Oxidative stress in chronic kidney disease is a multifactorial process which can be caused by an impairment of antioxidant mechanisms as well as an increased production of reactive oxygen species (ROS) [36, 37]. It was shown in rats that CKD is associated with a depressed superoxide dismutase activity parallel to an increase in NADPH oxidase expression [35]. Nrf2, a translation factor which controls expression of antioxidant genes, is a key player in resistance to oxidative stress and may represent a potential target in this process [38]. Thus, in rats with CKD, Nrf2 activity was markedly reduced beside an increase in biomarkers of oxidative stress and inflammation [34].

Recent controlled randomized clinical trials have demonstrated a beneficial effect of SGLT2-inhibitors in heart failure patients with and without diabetes regarding the occurrence of major cardiovascular endpoints [39–41]. Of note, SGLT2-inhibition was additionally associated with a slower progression of CKD in patients with and without diabetes [42, 43]. Aside from an improved glycemic control, the discussion about the beneficial cardiovascular and renal effects of SGLT2-inhibitors is widely focused on volume control via the induction of natriuresis and osmotic diuresis [44]. On a molecular level, SGLT2-inhibitors have been shown to reduce oxidative stress, which may suggest a benefit of these substances regarding vascular alterations in patients with or without CKD [45, 46]. Although the molecular mechanisms of SGLT2-inibitiors mediating the protective effects in vascular and kidney disease are still vastly unknown, they represent a promising novel target to treat patients with CAD und CKD.

Recently, interleukin 1 (IL-1) has emerged as a potential therapeutic target to contain inflammation in CKD. IL-1 is a cytokine which is activated by the NLRP3 inflammasome and that induces IL-6 which is independently associated with an increase in mortality in hemodialysis and pre-dialysis patients [47, 48]. Malnutrition is another common finding among CKD patients and was shown to influence circulating inflammation markers in a bidirectional way [49, 50]. In this context, it is of interest that on the basis of the available evidence, a “malnutrition inflammation atherosclerotic syndrome” has been proposed [50].

Chronic inflammation in CKD is a multifactorial condition and specific pharmacological targets to improve outcomes for patients are rare. IL-1β inhibition has shown promising results in animal models with different renal disorders [47, 51, 52]. In a randomized, double-blind placebo-controlled trial with over 10,000 patients, administration of canakinumab, a human monoclonal antibody that targets IL-1β, was associated with a significantly lower rate of recurrent cardiovascular events than placebo [53]. In a subgroup analysis, it was shown that inhibition of IL-1β is particularly promising regarding cardiovascular outcomes in CKD patients [47]. At present, however, canakinumab is not approved for this indication in the USA or Europe. Other anti-inflammatory substances with a broader mechanism of action have also shown promising results. In a recent placebo-controlled trial, colchicine was shown to significantly reduce the risk of ischemic cardiovascular events in patients with myocardial infarction [54]. To date, no such studies have been performed specifically in patients with CKD. In this patient population, safety concerns are of special importance since colchicine is partly eliminated by the kidney and is not removed through hemodialysis [55].

### Endothelial dysfunction

Accumulating evidence suggests that CKD promotes atherosclerosis and CAD by inducing damage to endothelial cells. Albuminuria as a consequence of glomerular damage was shown in several studies to correlate with elevated levels of von Willebrand factor, an indicator of endothelial dysfunction [56–58]. Furthermore, albuminuria and CKD were shown to be associated with microvascular endothelial dysfunction [59]. In order to control vascular tone, the endothelium synthesizes and releases the vasodilator nitric oxide (NO) from the amino acid L-arginine [60]. In addition, NO plays an important role in regulating vascular permeability, leukocyte adhesion and smooth muscle cell proliferation [61]. Therefore, a hallmark in endothelial dysfunction is a decrease in NO synthesis or bioavailability which is frequently observed in CKD patients [62, 63]. Amador-Martinez et al. were recently able to show in a rat model that cardiac alterations in CKD are partly promoted by an inactivation of endothelial nitric oxide synthase (eNOS) leading to reduced synthesis of NO [64]. L-arginine deficiency is a known factor leading to a decreased activity of eNOS and substitution of L-arginine was shown to positively affect cardiac alterations in rats with CKD [64].

Important mediators of endothelial dysfunction in CKD are uremic toxins such as asymmetric dimethylarginine (ADMA) and indoxyl sulfate (IS), which accumulate in CKD patients in parallel with declining renal function. ADMA disturbs endothelial function by competitive inhibition of eNOS and is closely associated with the presence and functional significance of CAD in CKD [65, 66]. Similarly, IS is involved in the pathogenesis of CRS through impairment of endothelial NO synthesis and, consecutively, endothelial proliferation in vivo and in vitro [67, 68]. Thus, the role of these uremic toxins may also extend into participating in the complex interrelationship of the heart and the kidney as a mediator of endothelial dysfunction.

Another recently described mechanism of endothelial dysfunction in CKD is driven by a change in the functional properties of LDL-cholesterol. Thus, carbamylation of lysine residues of the LDL-particle is primarily observed in CKD patients promoting endothelial dysfunction by increasing reactive oxygen species (ROS) production and eNOS uncoupling [69].

Hyperphosphatemia as a frequently observed finding among patients with CKD is known to be a major factor involved in the development of medial calcification in CKD (see below). In vitro and in vivo data available suggest a direct influence of elevated phosphate on endothelial function [70, 71]. An hyperphosphatemic milieu results in an impairment of angiogenesis, endothelial migration, and survival; a possible molecular mechanism for this finding could be the downregulation of annexin II [70].

### Vascular calcification in CKD

While atherosclerosis is characterized through vascular endothelial dysfunction progressing to vascular structural damage, the medial layer of blood vessels is affected differently in CKD [72]. In this layer of the vessel wall, the primary abnormality is VC [73]. VC is highly prevalent in CKD patients and even present in young adults with ESRD lacking typical cardiovascular risk factors such as hypertension or dyslipidemia [12–14]. VC manifesting in the coronary arteries impairs coronary flow reserve and is associated with a marked increase in adverse cardiac events and cardiovascular mortality [13, 15, 16]. VC occurs in two different phenotypes, medial and intimal calcification, differing in their pathogenesis [74, 75]. While intimal calcification is mainly inflammation-driven and closely associated to atherosclerotic plaques, medial calcification is considered to be the major form of VC in CKD [74, 76]. As a result of intimal VC, uremic patients present with heavily calcified atherosclerotic plaques whereas in the absence of CKD, plaques are more fibroatheromatous [77, 78]. Furthermore, increased deposition of CRP in plaques of CKD patients has been reported which may be an indicator for a higher inflammatory component [77, 78].

**Fig. 1:**
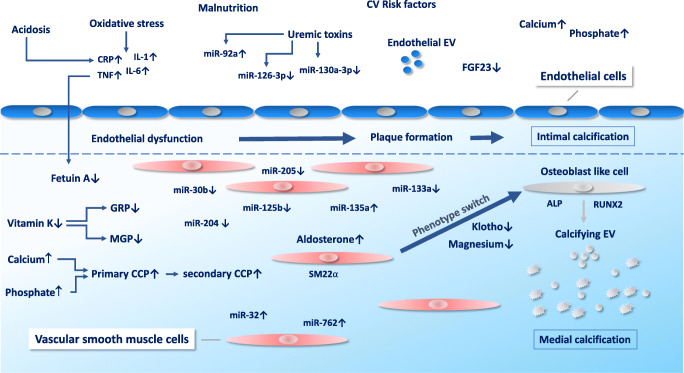
Intimal and medial calcification in CKD. CKD-specific risk factors are adding up to traditional cardiovascular risk factors and result in endothelial dysfunction and intimal calcification. A phenotype switch of vascular smooth muscle cells to osteoblast-like cells with a loss of smooth muscle cell markers like SM22a and expression of osteochondrogenic markers like Runt-related transcription factor 2 (RUNX2) and alkaline phosphatase (ALP) represents a crucial event in medial calcification. In this process, levels of miRs are altered which act as regulators of calcification in CKD. MGP matrix Gla protein, GRP Gla-rich protein, EV extracellular vesicles, CV cardiovascular, miR microRNA, CCP calciprotein particle

VC is a cell-mediated process similar to skeletal bone formation (Fig. 1). A crucial event is a phenotype switch of vascular smooth muscle cells (VSMC) to osteoblast-like cells which is induced through a number of different stimuli like hyperphosphatemia and hypercalcemia [27, 75, 79]. During the process of transdifferentiation of VSMC, a “reprogramming” is observed with a loss of SMC markers like SM22α and newly expression of osteochondrogenic markers like Runt-related transcription factor 2 (RUNX2), alkaline phosphatase (ALP), osteopontin and osteocalcin [27, 80, 81]. On a cellular level, VC shows similarities with cellular processes observed in senescence which underlines the theory that CKD is a risk factor for premature vascular aging [82, 83]. In the process of VC, VSMC-derived extracellular vesicles (EV) (formerly matrix vesicles) containing calcium phosphate crystals cause mineralization in the extracellular matrix (ECM) [84, 85, 86] .

In 2006, a work group of the Kidney Disease: Improving Global Outcomes (KDIGO) recommended the introduction of the term “chronic kidney disease-mineral and bone disorder” (CKD-MBD) to describe a syndrome consisting of abnormalities in bone and mineral metabolism as well as extra-skeletal calcifications including VC [87]. Mineral dysregulation and especially phosphate accumulation play a key role, since inorganic phosphate, known to mediate vascular calcification in a time- and dose-dependent manner, is frequently elevated in CKD patients [79]. Phosphate accumulation as a result of a declining renal clearance and secondary hyperparathyroidism were shown to induce an upregulation of osteogenic gene expression in vitro [76]. In this context, the activity of specific enzymes that regulate biomineralization such as tissue-nonspecific alkaline phosphatase (TNAP) becomes of interest. TNAP regulates extracellular pyrophosphate, an inhibitor of calcification and, accordingly, TNAP overexpressing mice showed a significant increase in medial calcification [88, 89]. Alterations in bone metabolism of CKD patients, or renal osteodystrophy, are a multifaceted disorder with adynamic bone disease as the most frequently observed form among patients on dialysis [90]. Adynamic bone disease is characterized by a low bone turnover and is known to be associated with VC in nondialysis CKD patients as well as in patients on hemodialysis [90–92]. A common finding among CKD patients is a deficiency of vitamin D which can lead to hypocalcemia. Low circulating levels of 25-hydroxyvitamin D are known to be associated with increased mortality in CKD patients as well as the development of VC [93, 94]. Vitamin D substitution may have beneficial effects on VC development in CKD patients, a potential mechanism for this finding is that vitamin D known suppresses calcification by downregulation of RUNX2 [95, 96]. However, dosage and indication for vitamin D substitution should be chosen wisely. Elevated concentrations of calcium and the calcium-phosphate product may markedly influence VSMC calcification [27, 97]. Excessive substitution of vitamin D or the administration of calcium containing phosphate binders can be associated with intermittent increases in serum calcium concentration [98]. Moreover, apoptotic or necrotic cells in the vessel wall can also mediate elevated levels of calcium [27]. In the presence of elevated phosphate levels, even small elevations of calcium were shown to act synergistically to promote vascular calcification [97]. Under these calcifying stimuli, calcium phosphate crystals are loaded onto EV by VSMC which nucleate in the ECM as hydroxyapatite [86].

Despite the fact that elevated phosphate levels are known to be associated with adverse cardiovascular outcomes in CKD patients, only few studies have examined clinical endpoints of interventions regarding phosphate control [99]. Meta-analyses of available randomized controlled trials regarding phosphate binding agents have demonstrated a lower all-cause mortality of patients receiving the non-calcium-based agent sevelamer compared to calcium-based binders while there was no significant difference regarding cardiovascular death [100, 101]. According to available data from clinical trials comparing phosphate binders to placebo in nondialysis CKD patients, the relevance of the administration of phosphate binders has not been proven in this population[102–103]. The “IMpact of Phosphate Reduction On Vascular End-points in Chronic Kidney Disease” (IMPROVE-CKD) study, a multicenter, randomized parallel-group trial, examined the impact of the non-calcium-based phosphate binder lanthanum carbonate on surrogate markers of cardiovascular disease compared with placebo [99]. Experimental background of that study was, among others, an in vitro study in rat aortic tissue which showed that the administration of iron citrate resulted in an anti-calcific effect by preventing or partially reverting high phosphate induced osteo-chondrocytic shift of ECM [105]. Treatment of CKD patients with lanthanum over 96 weeks however showed no significant difference to placebo regarding arterial stiffness and aortic calcification [106]. However, it is worth mentioning that in this nondialysis CKD population, the patients presented with normophosphatemia at baseline. The influence of a positive phosphate balance on this therapeutic approach needs to be further investigated [106].

In contrast to calcium and phosphate, magnesium has emerged to be a possible protector against VC. Clinical data suggest an inverse association between serum magnesium levels and the expression of VC in peritoneal- and hemodialysis patients [107, 108]. In vitro studies performed on VSMC were able to show a decreased formation of hydroxyapatite formation after exposing the cells to increased magnesium concentrations, a potential molecular mechanism for this finding is suggested to be an impairment of β-glycerophosphate induced ALP activity [109, 110]. In addition, data from experimental studies suggests magnesium to counteract VSMC transdifferentiation by impairing the expression of osteogenic transcription factors [111, 112]. Therefore, magnesium supplementation might present a potential therapeutic approach to battle VC and needs to be investigated in future clinical studies.

More recently, impairment of calcification inhibiting mechanisms, such as fetuin A, klotho, and the vitamin K-dependent matrix Gla protein (MGP) and Gla-rich protein (GRP) have emerged as participants in the complex multifactorial process of VC [113, 114]. These proteins act as inhibitors for the precipitation of calcium/phosphate crystals by forming the calciprotein particle (CPP). CPP exist in two different phenotypes, primary and secondary CPP, which differ in shape, function, and diameter [115]. In vitro, secondary CPP are capable to induce VSMC calcification, expression, and release of TNF-α and may thereby represent a promising new biomarker for VC and potential therapeutic target [115]. In addition to their suggested influence on atherosclerosis, both systemic inflammation and malnutrition are associated with progression of VC [116]. A potential mechanism for this finding is supposed to be mediated through decreases in fetuin A levels, which were shown in several studies to be associated with an increase in vascular calcification and cardiovascular mortality [117, 118].

Another key calcification inhibitor which is impaired in CKD patients is the klotho/FGF23 axis. The klotho gene was first described as an aging suppressor and encodes for a single-pass transmembrane protein which functions as a coreceptor for fibroblast growth factor 23 (FGF-23) [119, 120]. CKD is characterized by klotho deficiency and low levels of circulating klotho were shown to be associated with adverse renal outcome [120, 121]. In addition to being a biomarker for CKD, klotho plays a pathogenic role in CKD and especially in VC. Evidence suggests that klotho improves the phosphate metabolism by inducing phosphaturia and supresses thereby VSMC calcification [120]. In line with these findings, animal models of klotho substitution have shown positive effects regarding kidney injury and phosphaturia [122, 123]. Klotho substitution may represent a promising therapeutic approach to influence VC in CKD patients. FGF23 in contrast is known to be elevated in CKD patients and is a potent predictor of adverse cardiovascular outcomes [124]. However, FGF23 was shown to be independently associated with VC, its relevance as an therapeutic target remains subject of ongoing research [125]. In a rat model of CKD, FGF23 neutralization was shown to positively influence secondary hyperparathyroidism but accelerated hyperphosphatemia, vascular calcification, and mortality [126].

Interestingly, hyperaldosteronism, which is a frequent finding among patients with CKD, is also observed in klotho-deficient mice and is supposed to participate in VC [127, 128]. VSMC express the mineralcorticoid receptor which is sensitive for aldosterone and can be blocked by the competitive antagonist spironolactone [129]. Experimental data provide evidence that aldosterone leads to the expression of the type III sodium-dependent phosphate transporter PIT1, which leads to an increase of ALP activity and expression of ostegenic transcription factors [130]. In an animal model with klotho-deficient mice, this effect was ameliorated through the administration of spironolactone-associated with an enhanced survival of the animals [130]. However, until now, no data are available regarding the positive influence of spironolactone on VC in patients.

Subclinical vitamin K deficiency is frequently observed in CKD patients and can be due to malnutrition, dietary restrictions, or anticoagulant therapies with vitamin K antagonists [131, 132]. This leads to a reduced bioavailability of vitamin K-dependent proteins. MGP and GRP are proteins synthesized by vascular smooth muscle cells and known as inhibitors of tissue calcification, while GRP may also exhibit anti-inflammatory efficacy [114, 133, 134]. To be fully active, both require vitamin K-dependent posttranslational modification (gamma carboxylation) [135]. Both MGP or GRP deficiency are associated with early development of VC in vitro and in vivo and thus represent a molecular mechanism connecting vitamin K deficiency in CKD with VC [136, 137]. The pathophysiological importance of an impairment of calcifying inhibitors is impressively highlighted by the example of calciphylaxis. Calciphylaxis is a rare syndrome with a high mortality characterized by calcification and thrombotic occlusion of microvessels in subcutaneous adipose tissue which results in painful, ischemic skin lesions [138]. It shares a number of pathophysiological features with VC such as a reduction of carboxylated MGP, fetuin A deficiency, and the use of vitamin K antagonists as a risk factor [138]. Despite its synonym “calcific uremic arteriolopathy,” calciphylaxis is also reported in nonuremic patients without advanced renal impairment. In contrast to VC, which typically affects the aorta, coronary, and femoral arteries, calciphylaxis occurs in the microcirculation of subcutaneous adipose tissue [138]. Therefore, calciphylaxis represents a distinct entity of VC rather than a continuum of VC [138].

Since vitamin K deficiency represents a key feature in the development of VC, the effects of vitamin K substitution have been investigated. A recently published study comparing oral vitamin K2 substitution versus placebo in CKD patients showed no improvement in vascular stiffness or other measures of vascular health [139]. Vitamin K1 supplementation however is a promising and uncomplicated way to positively influence VC in CKD patients and, therefore, its effect on CV risk is under investigation in current clinical trials (Table 3) [135, 140]. Further support for an essential role of vitamin K in vascular calcification is provided by experimental and clinical studies with vitamin K antagonists suggesting adverse effects on cardiovascular outcomes[141–143].

**Table 3:** CKD-specific mediators of VC and potential therapeutic approaches. *MGP*, matrix Gla protein; *GRP*, Gla-rich protein; *miR*, microRNA; *RUNX2*, Runt-related transcription factor 2

Mediators VC in CKD	Potential therapeutic approach
Hyperphosphatemia	Administration of phosphate binders
Vitamin K deficiency	Vitamin K substitution
Klotho deficiency	Klotho substitution
RUNX2 expression in VSMC	Administration of miR-133a, miR-204, miR-205
Inflammation	Anti-inflammatory substances (canakinumab, colchicine)
Secondary hyperparathyroidism	FGF23 neutralization
Hypomagnesemia	Magnesium substitution
Hyperaldosteronism	Spironolactone
Vitamin D deficiency	Vitamin D substitution

### Extracellular vesicles as intercellular messengers in CKD and CAD

In the past years, circulating EV have emerged to play a key role in cardiovascular health and disease [72]. EV are membrane shed particles that consist of a lipid bilayer and are categorized into exosomes (30–100 nm), microvesicles (200–1000 nm), and apoptotic bodies (1–4 μm) with each category having its own formation process [144, 145]. EV play an important role in intercellular communication by transferring their bioactive cargos including proteins, lipids, and nucleic acids. This mechanism of signal transfer has evolved as an important regulator of cardiovascular health and disease [146, 147]. Circulating EV are released from their origin cell in consequence of certain stimuli. Circulating EV are known to mediate many physiological and pathophysiological mechanisms like inflammation and coagulation[148–150]. Endothelial cell damage and apoptosis are crucial steps in the pathophysiology of CVD and endothelial microvesicles have been shown to be elevated in patients with CVD compared to healthy control subjects. This has been described in patients with hypertension, CAD, acute coronary syndrome, and myocardial infarction[151–154]. We have previously shown that endothelial-derived EV promote endothelial cell repair by delivering functional miR-126 into recipient cells. This mechanism was shown to be altered under pathological hyperglycemic conditions [155]. Furthermore, elevated levels of endothelial- and platelet-derived EV have been reported in CKD patients compared to healthy controls [156, 157]. Endothelial cells incubated with IS show an increase in EV release and a significantly reduced angiogenesis in endothelial progenitor cells [158]. Increased levels of endothelial EV were shown to be an independent predictor of CV mortality in patients with ESRD [159]. In vitro, endothelial EV from plasma of patients with ESRD led to reduced endothelium-dependent relaxations and cyclic guanosine monophosphate (cGMP) generation [160]. In addition to these findings, endothelial-derived EV correlate with an impaired arterial function in patients with ESRD, demonstrated by an accelerated aortic pulse wave velocity and loss of flow-mediated dilation [160].

VSMC-derived EV play a crucial role in the early stages of development of VC [84–86]. EV produced by VSMC under physiological conditions do not contain calcium phosphate crystals and moreover transport calcification inhibitory proteins such as vitamin K-dependent MGP and fetuin-A [84, 161]. Under calcifying stimuli such as elevated phosphate levels as they are present in CKD patients, calcium phosphate crystals are loaded onto the EV [86]. It is important to mention that EV secretion by VSMC is not a pathological process itself. Thus, removal of exosomes from healthy serum promoted VC which underlines the importance of exosomes as calcifying inhibitors under certain circumstances [162]. In vitro, VSMC that were incubated with serum of CKD patients demonstrated increased calcification compared with controls. Serum of CKD from which EV had been removed showed a marked decrease in VSMC calcification [162]. Taken together, these findings indicate that, on a molecular level, EV act as mediators between CKD and CVD. Influencing the phenotype and cargo of EV derived from VSMC could therefore represent an interesting therapeutic target for VC in CKD patients.

### microRNAs as gene regulators of vascular alteration in CKD

MicroRNAs (miRNAs) play an outstanding role in post transcriptional gene regulation and can be transferred intercellularly through EV [163]. MiRNAs are small, noncoding nucleotides of about 20 basepaires that modulate different biological pathways in angiogenesis and apoptosis as well as diseases such as atherosclerosis and CKD [164, 165]. We have previously shown that atherosclerotic conditions promote the packaging of endothelial miR-92a-3p into endothelial microvesicles which regulates angiogenesis and may act as a potential regenerative messenger in intercellular communication [166]. Patients with diabetic nephropathy express a different exosomal miRNA profile than healthy subjects and upregulated miRNAs closely correlate with the degree of albuminuria [167]. A pilot study, comparing urinary and plasmatic miRNA-profiles of CKD patients with eGFR < 30 ml·min^−1^·1.73 cm^−2^ and patients with eGFR ≥ 30 ml·min^−1^·1.73 cm^−2^ showed 266 circulatory and 384 urinary miRNA that were differently expressed [168]. A number of upregulated miRNA in this study, including miR-130a-3p and miR-1825, target the TGFβ pathway [168]. Own data reveal that CKD patients exhibit significantly lower vesicular levels of vascular protective miR-126-3p and miR-130a-3p [169]. In vitro treatment with the model uremia toxin IS leads to a decreased packaging of the two miRNAs into EV through a hnRNPU-dependent sorting mechanism. This altered miRNA packaging was shown to be functionally relevant as it influences endothelial cell migratory capacity [169]. Another miRNA, miR-92a, which is relevant for endothelial function circulates in augmented levels in the peripheral blood of CKD patients. It correlates with levels of IS and is transported by endothelial microvesicles that originate from uremia-damaged endothelial cells [170]. In cultured endothelial cells, miR-92a induced inflammasome activation and thus mediated endothelial cell damage [170]. MiRNAs have been investigated as therapeutic targets to reduce CKD-associated atherosclerosis. Thus, in mice with renal injury, increased levels of miR-92a-3p were observed and inhibition of miR-92a-3p with a single injection miRNA inhibitors complexed to HDL significantly reduced atherosclerotic lesions [171]. Inhibition of these miRNAs significantly altered the TGFβ pathway and STAT3 trancriptional activity [171]. Furthermore, recent data support the concept that substitution of cardiovascular protective miRNAs may represent a potential treatment strategy. One such target investigated is miR-142-3p which is inversely correlated with carotid-femoral pulse wave velocity in humans as an indicator of vascular stiffness and is significantly decreased in patients with ESRD [172]. Also, in uremic mice with markedly reduced acetylcholine-induced relaxation, intravenous injection of synthetic mimic syn-mmu-mir-142-3p in order to restore bioavailability of miR-142-3p 2 days before sacrifice increased aortic relaxation of aortic rings to control levels [172].

VC is a cell-mediated process that requires genetic alterations which may at least in part be modulated through miRNAs [173]. RUNX2, a transcription factor which regulates osteoblast differentiation and VSMC calcification, represents a key target for miRs influencing VC. Recent data show protective miR like miR-133a, miR-204, and miR-205, as well as miR triggering VC like miR-32 influence RUNX2 by decreasing or inducing its expression [174–177]. Furthermore, miR-30b and miR-125b have recently been identified to protect against VC. Bone morphogenetic protein-2 (BMP-2) may promote vascular calcification by decreasing miR-30b and miR-30c to induce RUNX2 expression whereas upregulation of miR-30b attenuated VC in vivo and in vitro [178, 179]. In vitro, downregulation of endogenous miR-125b led to osteogenic transdifferentiation of VSMC and increased activity of alkaline phosphatase activity and matrix mineralization which was confirmed in ApoE knockout mice [180]. Osterix, an osteoblast transcription factor, may be a molecular target of miR-125b in this process [180]. In an in vitro biomineralization model, VSMC that have been transfected with miR-125b showed significantly lower osteocalcin expression than control VSMC [181]. These data are supported by the observation that decreased circulating levels of miR-125b in CKD patients is associated with a decline in renal function [182]. Recent studies have elucidated the potential use of miR-125b as a biomarker for VC in uremic patients. MiR-125b levels correlated significantly with VC severity as well as levels of fetuin-A and mediators of mineral bone disorder like osteoprotegerin and FGF-23 [181, 183]. In one study, high levels of serum osteoprotegerin and low serum miR-125b levels were able to synergistically enhance VC risk estimating ability [183]. Furthermore, miR-135a, miR-762, miR-714, and miR-712 targeting Ca^2+^-transporters in VSMC were shown in vitro and in vivo in a rat model to be overexpressed in the aortic media under calcifying conditions [184]. Recently, Fakhry et al. investigated potential miRs involved in VC by exposing rat aortic explants to high concentrations of phosphate and characterized the miR expression profile versus control samples [80]. In that study, five miRs were expressed differently compared to controls (miR-155, -200c, -322, -331, -708) 3 days after exposition to phosphate and five miRs (miR-328, -546, -301a, -409, -542) 6 days after exposition [80]. It is of interest that some of the altered miRs (miR-322, miR-155, miR-200c) are known to act by participating in inflammatory and osteoblastic processes [80]. Finally, Pan et al. were able to show that miR profiles in EV from mouse VSMC were significantly influenced through VC [185]. Taken together, miRs represent important regulators of VC in CKD and further understanding of single pathways will be crucial to develop molecular treatment targets.

In summary, cardiorenal syndrome, the combination of cardiovascular and chronic kidney disease, is a crucial challenge for modern medicine with rising numbers in incidence and prevalence. Vascular calcification represents an important link between CKD and cardiovascular mortality which is the leading cause of death among patients with impaired renal function. While numerous potential pathophysiological mechanisms have been uncovered in recent years, many details remain unknown, and as a consequence, therapeutic options to provide better outcomes for patients with VC are limited. A further understanding of molecular mechanisms and genetic targets involved in the complex process of VC is pivotal to develop novel therapeutic targets. However, more research is required to transfer promising experimental results into routine clinical practice. With a better understanding of the pathophysiological mechanisms involved in VC, in the future, additional therapeutic targets with a chance for further improvements in therapy may be identified.

## Data Availability

Not applicable.

## Abbreviations

ADMA: Asymmetric dimethylarginine
ALP: Alkaline phosphatase
BMP-2: Bone morphogenetic protein-2
CAD: Coronary artery disease
cGMP: Cyclic guanosine monophosphate
CKD: Chronic kidney disease
CRP: C-reactive protein
CPP: Calciprotein particle
CRS: Cardiorenal syndrome
CVD: Cardiovascular disease
ECM: Extracellular matrix
eNOS: Nitric oxide synthase
ESRD: End stage renal disease
EV: Extracellular vesicles
GRP: Gla-rich protein
IL-1: Interleukin-1
IL-6: Interleukin-6
IS: Indoxyl sulfate
MGP: Matrix Gla protein
miRNA: MicroRNAs
NO: Nitric oxide
ROS: Reactive oxygen species
RUNX2: Runt-related transcription factor 2
TNAP: Tissue-nonspecific alkaline phosphatase
TNF: Tumor necrosis factor
VC: Vascular calcification
VSMC: Vascular smooth muscle cells
